# Implementing the renewed vision for Primary Health Care in the Declaration of Astana: the time is now

**DOI:** 10.1017/S1463423619000719

**Published:** 2019-10-21

**Authors:** Hans Kluge, Edward Kelley, Yelzhan Birtanov, Pavlos N. Theodorakis, Shannon Barkley, Serzhan Aidossov, Jose M. Valderas

**Affiliations:** 1 Director, Division of Health Systems and Public Health, Regional Office for Europe, World Health Organization, Copenhagen, Denmark; 2 Co-Chair, Alma-Ata 40th Anniversary Technical Coordination Team, Geneva, Switzerland; 3 Director, Department of Health Systems and Safety, World Health Organization, Geneva, Switzerland; 4 Ministry of Healthcare of the Republic of Kazakhstan, Nur-Sultan, Kazakhstan; 5 Senior Advisor, Division of Health Systems and Public Health, European Centre for Primary Health Care, Regional Office for Europe, World Health Organization, Almaty, Kazakhstan; 6 Member, Alma-Ata 40th Anniversary Technical Coordination Team, Geneva, Switzerland; 7 Technical Officer, Department of Health Systems and Safety, World Health Organization, Geneva, Switzerland; 8 Advisor to the Ministry of Healthcare of the Republic of Kazakhstan, Nur-Sultan, Kazakhstan; 9 Managing Director of the Republican Centre for Health Development, Ministry of Healthcare of the Republic of Kazakhstan, Nur-Sultan, Kazakhstan; 10 Professor of Health Services & Policy Research Group, Exeter Collaboration for Academic Primary Care (APEx) and NIHR PenCLAHRC, University of Exeter, Exeter, UK; 11 Consultant, Department of Health Systems and Safety, World Health Organization, Geneva, Switzerland

The year 2018 marked the 40th anniversary of the 1978 Declaration of Alma-Ata (WHO and UNICEF, 1978). Four decades on from the first political commitment to primary health care (PHC), a deep consensus remains that the health and well-being of populations is most effectively, equitably and efficiently achieved through the PHC approach, making it a cornerstone of a sustainable health system for universal health coverage (UHC) and the health-related Sustainable Development Goals (Kluge *et al*., 2018a).

## Global conference

Events across the year culminated in the Global Conference on Primary Health Care: ‘From Alma-Ata towards universal health coverage and the Sustainable Development Goals’ in Astana, Kazakhstan, on 25 and 26 October 2018. More than 2000 individuals were convened by WHO, UNICEF and the Government of Kazakhstan for 2 days of a packed agenda (World Health Organization, 2018a). Participants represented member states, relevant entities of the United Nations system, donors, technical and financial partners, nongovernmental organizations, civil society, professional organizations and academia from nearly 150 Member States. Attendees came together to renew a commitment to placing PHC at the heart of achieving UHC, and the Sustainable Development Agenda embodied the new Declaration of Astana. Primary Heath Care Research was part of the agenda, too, with an exciting event ‘Agenda for research based on the renewed vision for Primary Health Care: an international perspective’ convened by a Primary Care academics in collaboration with the Alliance for Health Policy and World Organization of Family Doctors.

## Declaration of Astana

In adopting the new Declaration at the conference, Member States reaffirmed the original principles and values highlighted in the Alma-Ata Declaration, as well as their commitment to the fundamental right of every human being to the enjoyment of the highest attainable standard of health without discrimination. The new Declaration calls for the mobilization of all stakeholders – health professionals, academia, patients, civil society, local and international partners, agencies and funds, the private sector and faith-based organizations to focus their efforts around the three main elements of PHC (World Health Organization, 2018b):

1. meeting people’s needs through comprehensive and integrated health services (including promotive, protective, preventive, curative, rehabilitative and palliative) throughout the entire life course, prioritizing primary care and essential public health functions;
2. systematically addressing the broader determinants of health (including social, economic and environmental factors, as well as individual characteristics and behaviours) through evidence-informed policies and actions across all sectors; and
3. empowering individuals, families and communities to optimize their health as advocates for policies that promote and protect health and well-being, as codevelopers of health and social services and as self-carers and caregivers.

## Vision and operational framework

Two key documents provided background for the Declaration: A Vision for Primary Health Care in the 21st Century (World Health Organization, 2018c) and Primary Health Care: Transforming Vision into Action – Operational Framework (World Health Organization, 2018d). The vision document proposes a set of levers that would help countries accelerate progress across the three components of PHC. The levers are separated into ones that primarily function at the policy level: political commitment and leadership; governance and policy frameworks, adequate funding and equitable allocation of resources and ones that primarily function at the operational level: engagement of community and other stakeholders to jointly define problems and solutions and prioritize actions, models of care that prioritize primary care and public health functions; ensuring the delivery of high-quality and safe health-care services; engagement with private sector providers, PHC workforce; physical infrastructure and appropriate medicines; products and technologies; digital technologies; purchasing and payment systems; PHC-oriented research; monitoring and evaluation, although the two levels are interdependent and most levers have elements that are policy and elements that are operational.

The operational framework, which underwent global public consultation, proposes how these levers can be implemented. The objective is to provide a framework for countries to prioritize, select and customize specific actions based on their social and economic development, degree of PHC orientation and health status. Two levers related to research will be of particular interest for the audience of the journal and will help exemplify the scope of the operational framework. The first one considers PHC-oriented research and knowledge management, including dissemination of lessons learned, as well as the use of the knowledge to accelerate scale-up of successful approaches. Based on the challenges that PHC research is currently facing (Kluge *et al*., 2018b), this lever considers actions at four levels: policy, for example, earmarking research funding in budgets for PHC programs and policies; operational and implementational, for example, supporting the development of primary care research networks; people and communities, for example, advocating public and patient involvement in research questions, study design and conduct, and dissemination; and global and regional partnerships, for example, promoting cross-national and regional networks of PHC research centers. Another lever linked to research considers monitoring and evaluation and focuses on well-functioning health information systems that generate reliable data and support their use for improved decision-making from local to global level.

## Need to act: the time is now

Another outcome of the conference was the identification of the critical need for international organizations and Member States to align implementation efforts with one another, and with existing initiatives. International organizations need to agree on modalities for collaboration and standard operating procedures. These should outline how to work together at both global and country levels, using existing, nationally led country coordination mechanisms contextualized by country, and including specific metrics that will define success (for both the partnerships as well as PHC health systems performance). A set of international partners is already working on implementing the vision of the Declaration. Moreover, the Government of the Republic of Kazakhstan requested from the Director General at the January 2019 WHO Executive Board Meeting in Geneva support for Member States, as appropriate, in strengthening PHC, including on the implementation of the vision and commitments of the Declaration of Astana in coordination with all relevant stakeholders, and to develop, in consultation with Member States by the 73rd World Health Assembly (2020), an ‘Operational Framework for Primary health care’, to be taken fully into account in the WHO program of work and budget to strengthen health systems and support countries in scaling-up national implementation efforts on PHC.

The previous months have seen an impressive collaborative effort to renew commitment for PHC and a shared understanding of what is needed for PHC to be a truly transformative approach that delivers health and well-being for all. It is imperative to translate the renewed vision into action. The time is now!
